# Global Distribution of *Shigella sonnei* Clones

**DOI:** 10.3201/eid1710.101486

**Published:** 2011-10

**Authors:** Ingrid Filliol-Toutain, Chien-Shun Chiou, Caterina Mammina, Peter Gerner-Smidt, Kwai-Lin Thong, Dac Cam Phung, Mariana Pichel, Reza Ranjbar, Amy Gassama Sow, Kara Cooper, Efrain Ribot, Norma Binsztein, Shiu-Yun Liang

**Affiliations:** Author affiliations: Institut Pasteur, Paris, France (I. Filliol-Toutain);; Chung Shan Medical University, Taichung, Taiwan (C.-S. Chiou);; Centers for Disease Control, Taipei, Taiwan (C.-S. Chiou, S.-Y. Liang);; University of Palermo, Palermo, Italy (C. Mammina);; Centers for Disease Control and Prevention, Atlanta, Georgia, USA (P. Gerner-Smidt, K. Cooper, E. Ribot);; University of Malaya, Kuala Lumpur, Malaysia (K.-L. Thong);; National Institute of Hygiene and Epidemiology, Hanoi, Vietnam (D.C. Phung);; Instituto Nacional de Enfermedades Infecciosas, Buenos Aires, Argentina (M. Pichel, N. Binsztein);; Molecular Biology Research Center, Baqiyatallah University of Medical Sciences, Tehran, Iran (R. Ranjbar);; Institut Pasteur de Dakar, Dakar, Senegal (A. Gassama Sow)

**Keywords:** Shigella sonnei, molecular epidemiology, global distribution, clones, bacteria, dispatch

## Abstract

To investigate global epidemiology of *Shigella sonnei*, we performed multilocus variable number tandem repeat analysis of 1,672 isolates obtained since 1943 from 50 countries on 5 continents and the Pacific region. Three major clonal groups were identified; 2 were globally spread. Type 18 and its derivatives have circulated worldwide in recent decades.

*Shigella sonnei* is the most commonly isolated species among the 4 *Shigella* species in industrialized countries (*1**,**2*). Transmission of *S. sonnei* across geographic boundaries is frequently linked to international travel and cross-border food trade (*3**,**4*). *S. sonnei* is a monomorphic organism and therefore requires a highly discriminative sequence-based method for investigating its clonal structure and the geographic distribution of clones.

A total of 26 variable number tandem repeats (VNTRs) have been used to type *S. sonnei* isolates (*5*). Because VNTRs have a wide range of variability, they are useful markers for investigating clonal relationships among strains that have evolved over different times (*6*). In this study, we analyzed 1,672 *S. sonnei* isolates obtained since 1943 from 50 countries on 5 continents (Africa, Asia, Europe, North America, and South America) and the Pacific region by multilocus VNTR analysis (MLVA) to investigate the global epidemiology of *S. sonnei*.

## The Study

Isolates were obtained from 50 countries on 5 continents and the Pacific region (Table A1). Of these isolates, 31 were obtained during 1943–1983 (from Cameroon, Denmark, France, Senegal, and Sweden) and 1,641 were obtained during 1994–2008 from 48 countries. Isolates were lyophilized, kept in stab culture medium, or stored in 15%–20% glycerol at –75°C for long-term storage. The isolates were not repeatedly subcultured before this study. MLVA26, an MLVA assay based on analysis of 26 VNTRs, classified the 1,672 isolates into 620 MLVA26 types. With only 2 exceptions, no common MLVA26 type was shared among isolates from different countries. One isolate from Malaysia (1999) and 2 isolates from Vietnam (2006) shared a common MLVA26 type, and an isolate from Chad and an isolate from France (both isolated in 2007) shared a common MVLA26 type.

The high resolving power of MLVA is primarily caused by highly diverse VNTRs (*6*). Clustering analysis of the MLVA26 types using a minimum spanning tree (MST) algorithm grouped the 1,672 isolates into 3 large clusters (A, B, and C), 1 small cluster (D), and 1 singleton (E). Each cluster was defined to include MLVA26 types differing at <7 loci among the 26 loci. The 3 large clusters displayed distinct allelic diversity features. Eight loci (SS1, SS3, SS6, SS9, SS10, SS11, SS12, and SS23) had Simpson diversity values >0.5 for >1 of the 3 large clusters (Table). Differences in diversity values >0.3 among the 3 clusters were observed for 9 of the 26 VNTRs. The largest difference was in 2 hypervariable VNTRs (SS1 and SS6). These 2 VNTRs displayed a high degree of allelic diversity in cluster A, but were invariant in cluster B. SS1 was invariant but SS6 displayed a high degree of diversity in cluster C.

**Table:** Allelic diversity and range of repeats of VNTRs for 3 major *Shigella sonnei* clonal groups*

VNTR	Repeat unit, bp	No. alleles	Clonal group A, n = 1,382			Clonal group B, n = 75			Clonal group C, n = 212	
			Allelic diversity†	Range of repeats		Allelic diversity	Range of repeats		Allelic diversity	Range of repeats
SS1	7	16	0.71	1–16		0	1		0	1
SS2	9	3	0.02	2–3		0	2		0.04	1–3
SS3	7	30	0.88	2–29		0.90	2–34		0.82	2–28
SS4	7	3	0.01	2–6		0	2		0.10	2–3
SS5	7	3	0.08	2–4		0.03	2–3		0	3
SS6	7	29	0.85	2–31		0	2		0.86	4–30
SS7	7	3	0.49	2–3		0	2		0.02	2–3
SS8	60	3	0	1		0.10	1–2, 330‡		0.45	1–2
SS9	6	16	0.65	2–18		0.83	5–18		0.77	2–18
SS10	6	9	0.57	2–10		0.46	3–8		0.53	0, 3–8
SS11	6	8	0.63	2–9		0.64	2–8		0.65	3–9
SS12	9	5	0.01	2–3		0.58	2–6		0.18	2–4
SS13	6	5	0.19	2–6		0.03	0, 2		0.07	2–4
SS14	9	4	0.01	2–3		0	2		0.37	2–6
SS15	6	3	0.04	2–3		0.21	3–4		0.02	2–3
SS16	17	3	0.07	1–2		0	2		0.06	1–3
SS17	6	2	0	0, 2		0.15	2–3		0.04	2–3
SS18	5	3	0.01	2–3		0	2		0.30	2–4
SS19	5	2	0.03	2–3		0.23	2–3		0.03	2–3
SS20	40	2	0.04	1–2		0	1		0	1
SS21	18	3	0.01	0, 1–2		0.12	1–2		0.24	1–4
SS22	11	2	0.01	1–2		0.03	1–2		0	1
SS23	16	8	0.03	0, 2–6		0.55	0, 2–5		0.69	0, 2–10
SS24	168	2	0.06	0, 1–2		0.03	0, 1		0.05	1–2
SS25	135	2	0.01	1–2		0.03	1–2		0.41	1–2
SS26	101	5	0.01	2–5		0.03	1–5		0	4

*VNTR, variable number tandem repeat. †Simpson's diversity index = 1 – Σ(n/N)^2^. ‡Allele 330 for SS8, which has an imperfect repeat, is designated by the size of amplicon instead of the number of repeats.

Of 1,672 isolates, 66% (1,100) were obtained from patients who acquired infections in Taiwan. Most isolates belonged to an insertion element IS1 interspacer 1 clone (*6**,**7*) that had slightly lower diversity values for some VNTRs than diversity values for total isolates obtained from a panel of 620 isolates representing the 620 MLVA26 types. However, a large number of clonal isolates from Taiwan did not affect relative magnitudes of diversity of the 26 VNTRs.

Although highly variable VNTRs are useful markers in distinguishing closely related strains, they are less useful for investigating clonal relationships among strains that have evolved over time (*6*). MLVA18 profiles, which excluded the data of 8 highly variable VNTRs (SS1, SS3, SS6, SS9, SS10, SS11, SS12, and SS23) from the 26-locus panel, were used to investigate the clonal structure of the isolates. On the basis of 18-locus profiles, 105 MLVA18 types were identified.

A simplified MST was created by analysis of a subset of 200 isolates selected by obtaining 1 MLVA18 type among those identified in each of 50 countries. As shown in the MST (Figure), cluster A was further divided into subclusters A1 and A2 and singleton A3, and cluster C was divided into subclusters C1 and C2.

**Figure:**
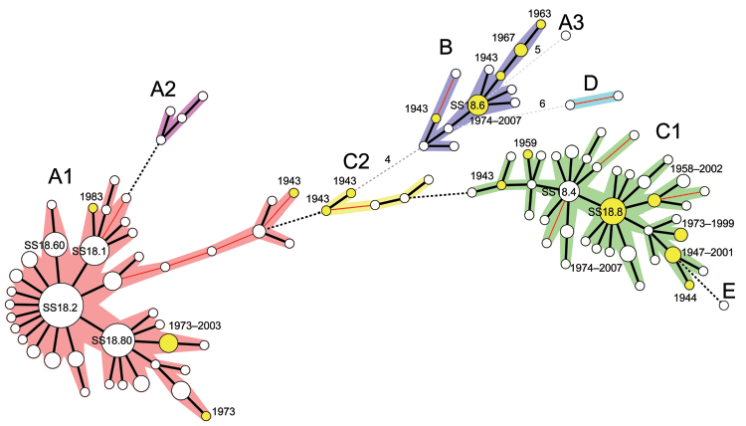
Clonal structure of 200 *Shigella sonnei* isolates. These isolates, representative of the 1,672 isolates analyzed in this study, were selected by obtaining 1 isolate for 1 multilocus variable number tandem repeat analysis (MLVA) 18 type from those identified in each of 50 countries on 5 continents (Africa, Asia, Europe, North America, and South America) and the Pacific region. The tree was constructed by using MLVA18 profiles and a minimum spanning tree algorithm. Circle size is proportional to the number of countries detected with the MLVA18 type. Genotypes in yellow indicate isolates obtained in the early period (1943–1983). A cluster or subcluster containing >2 genotypes differing at <2 loci is indicated by the 5 other colors. Distances of 1 locus between 2 closest genotypes are indicated by thick black lines, distances of 2 loci are indicated by thin red lines, distances of 3 loci are indicated by black dashed lines, and distances >4 loci are indicated by grey dashed lines. Numbers of different loci are indicated.

Cluster A consisted of 46 MLVA18 types, which represented 1,382 isolates obtained since 1943 in 40 countries on 5 continents and the Pacific region (Table A1). Several MLVA18 types within cluster A were widespread. SS18.2 was detected in 23 countries on 5 continents. SS18.2 had 14 single-locus variants (SLVs) at which genotypes differed only at 1 of the 18 loci; 3 (SS18.80, SS18.1, and SS18.60) of the SLVs were detected in 13, 10, and 7 countries, respectively. SS18.2 and its SLVs represented 1,290 isolates obtained in during 1995–2008 from 38 countries on 5 continents. Four MLVA18 types were detected in samples obtained in 1943–1983. These isolates shared identical MLVA18 profiles or differed at 1–2 loci from recently obtained isolates. Subcluster A2 consisted of 4 MLVA18 types found in isolates from Argentina only. Singleton A3, which was found in isolates obtained in Vietnam in 2008, was distantly separate from subclusters A1 and A2.

Cluster B consisted of 12 MLVA18 types representing 75 isolates, which were obtained in 8 countries in Africa, Asia, and Europe. SS18.6 had the highest number of SLVs in cluster B and was detected in 5 countries in Asia and Africa. Five types were detected in isolates obtained in 1943–1974; they shared identical MLVA18 profiles or differed at 1–2 loci from recently recovered isolates.

Cluster C was relatively diverse; it consisted of 44 MLVA18 types, which represented 212 isolates obtained in 21 countries on 5 continents and the Pacific region. SS18.8 had the highest number of SLVs and was found in isolates obtained during 1974–2007 in 8 countries on 5 continents. SS18.4, the largest SLV of SS18.8, was found in isolates from 5 countries on 5 continents. Subcluster C2 consisted of isolates from Argentina obtained in 2002 and Sweden and Denmark in 1943. These isolates emerged in 1943–1974 and were genetically similar to recently obtained isolates. Clusters (clonal groups) A and C were globally spread, and clonal group B was found in countries in Africa, Asia and Europe only.

Cluster D contained 2 isolates obtained in French Guiana and Senegal in 2003. The isolate for singleton E was obtained in Malaysia in 1999.

## Conclusions

Genetic analysis using MLVA presented a simple clonal structure for 1,672 *S. sonnei* isolates obtained since 1943 from 50 countries on 5 continents and the Pacific region. Three large clonal groups were identified; they displayed distinct allelic diversity features, particularly for 2 hypervariable VNTRs (SS1 and SS6). Clonal groups A and C were globally spread. One MLVA18 type (SS18.2) and several of its SLVs were widely distributed over 5 continents in the past 10 years.

## Appendix

**Table A1:** Geographic distribution of *Shigella sonnei* clones*

Continent and country	Year isolated	No. isolates	MLVA18 type (no. isolates)
Africa			
Algeria	2006–2007	2	Cluster A: SS18.60 (1), 80 (1)
Burkina Faso	2006	1	Cluster A: SS18.80 (1)
Cameroon	1973, 2007	2	Cluster A: SS18.2 (1); cluster C: SS18.102 (1)
Central African Republic	2007	1	Cluster A: SS18.60 (1)
Chad	2007	1	Cluster A: SS18.1 (1)
Egypt	2000, 2002, 2007	3	Cluster A: SS18.2 (2), 80 (1)
Côte d’Ivoire	2001	1	Cluster A: SS18.2 (1)
Kenya	2003, 2007	2	Cluster A: SS18.45 (2)
Madagascar	1998–2007	6	Cluster A: SS18.1 (1), 2 (1); cluster B: SS18.6 (1); cluster C: SS18.4 (1), 8 (2)
Mali	2006–2007	2	Cluster A: SS18.60 (2)
Mayotte	2004	1	Cluster C: SS18.8 (1)
Morocco	2001–2007	6	Cluster A: SS18.1 (2), 2 (1), 42 (1), 87 (1); cluster C: SS18.97 (1)
Nigeria	2007	1	Cluster A: SS18. 80 (1)
São Tomé and Principe	2008	1	Cluster A: SS18.58 (1)
Senegal	1967–2006	52	Cluster A: SS18.2 (36), 14 (6), 15 (5), 59 (1), 66 (1); cluster B: SS18.39 (1); Cluster C: SS18.90 (1); cluster D: SS18.19 (1)
Tanzania	2004	1	Cluster C: SS18.4 (1)
Tunisia	2006	1	Cluster A: SS18.15 (1)
Asia			
Myanmar	2007	1	Cluster A: SS18. 2 (1)
Cambodia	2004–2008	8	Cluster A: SS18.2 (1), 3 (5), 64 (1), 65 (1)
People’s Republic of China	2003–2007	6	Cluster A: 2 (4), 7 (1), 80 (1)
India	2000, 2007	3	Cluster A: SS18.1 (2), 55 (1)
Indonesia	2003–2008	16	Cluster A: SS18.60 (15), 85 (1)
Iran	2002–2003	52	Cluster A: SS18.1 (21), 2 (25), 62 (1), 63 (1), 66 (2), 80 (2)
Israel	1996, 2003, 2007	3	Cluster A: SS18.2 (3)
Malaysia	1994–2004	65	Cluster A: SS18.1 (8), 2 (10), 71 (1); cluster B: SS18.6 (17), 35 (1), 36 (1), 38 (1); cluster C: SS18.8 (6), 12 (9), 13 (3), 16 (1), 81 (2), 82 (2), 99 (2); singleton E: SS18.23 (1)
Nepal	2007	2	Cluster A: SS18.59 (1), 60 (1)
Pakistan	2001	1	Cluster C: SS18.8 (1)
The Philippines	2005	1	Cluster B: SS18.37 (1)
Taiwan	1996–2008	1,100	Cluster A: SS18.1 (704), 2 (203), 3 (23), 7 (1), 10 (19), 46 (1), 52 (1), 53 (1), 60 (1), 65 (10), 66 (1), 69 (1), 80 (2); cluster B: SS18.6 (22); cluster C: SS18.4 (61), 5 (29), 13 (6), 32 (1), 33 (1), 34 (1), 50 (1), 83 (2), 86 (1), 88 (1), 91 (1), 93 (2), 95 (1), 100 (1), 102 (1)
Thailand	2004, 2007	3	Cluster A: SS18.2 (2), 80 (1)
Turkey	2002	1	Cluster A: SS18.2 (1)
Vietnam	2004–2008	62	Cluster A: SS18.2 (28), 3 (7), 7 (1), 20 (1), 58 (1), 65 (2), 80 (6); cluster B: SS18.6 (15); cluster C: SS18.96 (1)
Europe			
Denmark	1943	1	Cluster C: SS18.21 (1)
France	1943–2007	73	Cluster A: SS18.1 (5), 2 (17), 26 (1), 41 (1), 54 (1), 58 (1), 59 (1), 60 (3), 61 (1), 67 (1), 68 (1), 80 (4); cluster B: SS18.6 (1), 28 (1), 29 (1), 30 (1), 31 (1), 39 (7), 40 (1); cluster C: SS18.4 (2), 8 (5), 9 (7), 16 (4), 79 (1), 83 (1), 89 (1), 92 (1), 94 (1)
Greece	2007	1	Cluster A: SS18.2 (1)
Italy	2001–2007	70	Cluster A: SS18.2 (48), 42 (2), 59 (1), 80 (8); cluster C: SS18.9 (8), 79 (1), 101 (1), 103 (1)
Poland	2006	1	Cluster A: SS18.1 (1)
Spain	2007	1	Cluster A: SS18.2 (1)
Sweden	1943–1947	8	Cluster A: SS18.25 (2); cluster B: SS18.27 (2); cluster C: SS18.9 (1), 22 (1), 104 (1), 105 (1)
North America			
Cuba	2003	1	Cluster A: SS18.80 (1)
Guatemala	2007	1	Cluster C: SS18.16 (1)
Haiti	2006	1	Cluster C: SS18.8 (1)
Mexico	2007	1	Cluster A: SS18.14 (1)
United States	1995–2007	49	Cluster A: SS18.2 (9), 14 (5), 26 (16), 47 (4), 49 (2), 51 (1), 57 (1); Cluster C: SS18.8 (1), 11 (3), 43 (3), 44 (2), 70 (2)
South America			
Argentina	2001–2007	48	Cluster A: SS18.1 (10), 2 (4), 48 (1), 58 (1), 66 (2), 72 (2), 73 (1), 74 (1), 75 (2), 76 (2), 78 (1), 80 (9); cluster C: SS18.4 (4), 5 (1), 17 (4), 24 (1), 56 (1), 93 (1)
Brazil	2006	1	Cluster C: SS18.98 (1)
French Guiana	1998, 2000, 2003, 2006	4	Cluster A: SS18.2 (3); cluster D: SS18.18 (1)
Venezuela	2007	1	Cluster C: SS18.8 (1)
Pacific			
New Caledonia	1997	1	Cluster A: SS18.77 (1)
Tahiti	2007	1	Cluster C: SS18.84 (1)

*MLVA, multilocus variable number tandem repeat.
